# The impact of lymphadenectomy on the survival of patients with stage I ovarian clear cell carcinoma

**DOI:** 10.3389/fonc.2024.1425214

**Published:** 2024-11-14

**Authors:** Tingting Li, Chunyan Tan, Sixia Xie, Hongjing Wang

**Affiliations:** ^1^ Department of Gynecology and Obstetrics, West China Second University Hospital, Sichuan University, Chengdu, Sichuan, China; ^2^ Key Laboratory of Birth Defects and Related Diseases of Women and Children, Sichuan University, Ministry of Education, Chengdu, Sichuan, China

**Keywords:** lymphadenectomy, the number of lymph nodes, ovarian clear cell carcinoma, survival, prognosis

## Abstract

**Objective:**

To assess the impact of lymphadenectomy on the survival of patients with stage I ovarian clear cell carcinoma (OCCC).

**Methods:**

The records of 93 patients with stage I OCCC treated between January 2012 and December 2019 were reviewed retrospectively. The relationships between survival outcomes and the number and region of removed lymph nodes (LNs) were assessed, and the independent prognostic factors were analyzed.

**Results:**

The median number of LNs resected in 93 patients was 24. These patients were divided into two groups based on the median number; overall survival (OS) and recurrence-free survival (RFS) differed significantly between the two groups. Patients were also grouped by the region of the LNs: the pelvic lymph node dissection (PLND) and PLND and para-aortic. Moreover, no differences in OS or RFS were observed between the two groups. Cox regression analysis demonstrated that the number of removed LNs was a significant and independent prognostic factor for poor RFS.

**Conclusion:**

This study exhibited that the number of removed LNs, as an important measure of adequate lymphadenectomy for stage I OCCC, contributed to improved RFS and OS. An independent prognostic factor for stage I OCCC was the number of dissected LNs.

## Introduction

Epithelial ovarian carcinoma (EOC), the seventh most commonly diagnosed cancer globally among women, is the most lethal gynecological malignancy (1, 2). Ovarian clear cell carcinoma (OCCC), a morphologically and biologically distinct neoplasm, comprises 5%–30% of all ovarian carcinomas (3). When OCCC has a favorable prognosis, it often presents at an early stage. The two independent prognostic factors are the International Federation of Gynecology and Obstetrics (FIGO) stage and response to chemotherapy (4). The primary treatment recommended for patients with OCCC is the standard surgical staging procedure or cytoreduction. However, the response rate to platinum-based chemotherapy is only 20%–50% for OCCC (5). Complete surgical staging may be critical for successful OCCC treatment owing to its chemo-resistant nature. Complete surgical staging, including pelvic and para-aortic lymphadenectomy, is recommended for patients with OCCC (6). However, the therapeutic significance of systematic lymphadenectomy in OCCC is debatable (7–16). The impact of lymphadenectomy on early-stage OCCC has been widely assessed (7–10). This study investigated the influence of lymphadenectomy in patients with stage I OCCC.

## Materials and methods

### Patient population

This retrospective study was conducted between January 2012 and December 2019 at the Department of Obstetrics and Gynecology, West China Second Hospital of Sichuan University. The approval for the study was obtained by the hospital ethics committee. The inclusion criteria were as follows: 1) patients diagnosed with primary pure OCCC, 2) those who underwent complete surgical staging [hysterectomy, bilateral salpingo-oophorectomy, omentectomy, peritoneal biopsies, cytology, pelvic lymph node dissection (PLND), and/or para-aortic lymph node dissection (PAND)], and 3) those diagnosed with FIGO stage I disease confirmed by pathological results after surgery. The exclusion criteria were as follows: 1) patients with mixed ovarian pathology, two primary sites (ovary and uterus), or other gynecological malignancies; 2) those with apparent FIGO stage I disease with nodal involvement; and 3) patients whose medical records were unavailable. A total of 93 patients were enrolled. Comprehensive surgical staging was performed, and no residual tumors were noted after surgery. Most patients received adjuvant chemotherapy post-surgery, except for those with FIGO stage IA disease or who refused adjuvant chemotherapy. Once the patient fulfilled the inclusion criteria and lacked the exclusion criteria, medical history, surgical and pathological data, and postoperative treatment and follow-up data were collected continuously.

### Data collection and patient groups

The clinicopathological data, including the age of the patients, FIGO stage, surgical approach followed, diameter of the tumor, number of removed lymph nodes (LNs), presence of coexisting endometriosis, presence of ascites, peritoneal cytology, rupture of capsule, restaging, lymphocyst, adjuvant chemotherapy, recurrence, recurrence-free survival (RFS), and overall survival (OS), were obtained from medical records. When the recurrence site was specified, it was surveyed in the patients. Missing data were collected during follow-up telephonic interviews. RFS was defined as the time elapsed between the date of operation and that of recurrence or the date of the last follow-up. OS was defined as the time elapsed from the date of operation to the date of death or the date of the last follow-up for living patients.

Based on the number of removed LNs, patients were divided into two groups, with the median number of nodes resected (24) serving as the cutoff. The two groups were defined as <24 and ≥24 LNs removed. Moreover, based on the regions where LN dissection was performed, either the pelvic region only or both the pelvic and para-aortic regions, patients were grouped.

### Statistical analyses

Pearson’s χ^2^ tests or Fisher’s exact tests were used to compare the clinical and pathological factor characteristics for categorical data, independent sample t-tests were used for normally distributed continuous data, and non-parametric tests were used for non-normal distribution data. The Kaplan–Meier method was used to determine OS and RFS. The log-rank test was used to compare prognostic factors for univariate analysis and Cox proportional hazards modeling for univariate and multivariate analyses. A *p*-value of <0.05 indicated statistical significance, and all *p*-values reported were two-sided. SPSS (IBM SPSS Statistics for Windows, Version 29.0) was used to perform the statistical analyses.

## Results

### Patient characteristics

**Table 1** presents the clinical characteristics of the patients based on the number of removed LNs. A total of 93 patients were included. The median number of removed LNs was 24. Among these patients, 48 (51.6%) and 45 (48.4%) had ≥24 and <24 LNs removed, respectively. The median age was 49 (26–67) years. The FIGO stage was IA and IC in 42 (45.2%) and 51 (54.8%) patients, respectively. Of these patients with FIGO stage I, 47 (50.5%) underwent adjuvant computed tomography/magnetic resonance imaging before the operation, 19 (45.2%) were FIGO stage IA, and 28 (54.9%) were FIGO stage IC. The numbers of patients with a tumor diameter ≤15 cm and >15 cm were 67 (72.0%) and 26 (28.0%), respectively. Fifty-one (54.8%) patients had coexisting endometriosis, whereas 42 (45.2%) patients did not. Of the 93 patients, only 33 (35.6%) had ascites. The peritoneal cytology in 86 (92.5%) patients was negative, while it was positive in six (7.5%) patients. Moreover, the tumor capsule in 48 (51.6%) patients was ruptured, while it was not ruptured in 45 (48.4%) patients. Regarding the surgical approach, 71 (76.3%) patients underwent laparotomy, while only 22 (23.7%) patients underwent laparoscopy owing to the size of the tumors. Although 72 (77.4%) patients underwent both PLND and PAND, 21 (22.6%) underwent only PLND. The most common complication after lymphadenectomy was lymphocyst. In our study, 44 (47.3%) patients had lymphocysts. Of the two patients who developed lymphocysts with infection, one underwent incision and drainage of the lymphocyst. There were 49 (52.7%) patients who did not have lymphocysts. A single surgery was performed in 66 (71.0%) patients, while two surgeries were performed in 27 (29.0%) patients. The patients with incomplete staging of the previous surgery or requirement of the expansion of the surgical scope after the initial surgery underwent restaging surgery. Among these, 87 (93.5%) patients received chemotherapy, while six (6.5%) did not receive chemotherapy.

**Table 1:** Patient characteristics according to the number of removed lymph nodes.

Characteristics	Total N = 93	≥24 LNs removed N = 48	<24 LNs removed N = 45	*p*-Value
Age (years)				0.365
Median (range)	49 (26–67)	48 (26–67)	40 (26–66)	
FIGO stage				0.484
IA	42 (45.2%)	20 (41.7%)	22 (48.9%)	
IC	51 (54.8%)	28 (58.3%)	23 (51.1%)	
Tumor diameter				0.114
≤15 cm	67 (72.0%)	38 (79.2%)	29 (64.4%)	
>15 cm	26 (28.0%)	10 (20.8%)	16 (35.6%)	
Coexisting endometriosis				0.846
Yes	51 (54.8%)	26 (53.2%)	25 (55.6%)	
No	42 (45.2%)	22 (45.8%)	20 (44.4%)	
Accompanied by ascites				0.080
Yes	33 (35.5%)	13 (27.1%)	20 (44.4%)	
No	60 (64.5%)	35 (72.9%)	25 (55.6%)	
Peritoneal cytology				0.275
Negative	86 (92.5%)	43 (89.6%)	43 (95.6%)	
Positive	7 (7.5%)	5 (10.4%)	2 (4.4%)	
Capsule rupture				0.611
Yes	48 (51.6%)	26 (54.2%)	22 (48.9%)	
No	45 (48.4%)	22 (45.8%)	23 (51.1%)	
Surgical approach				0.075
Laparotomy	71 (76.3%)	33 (68.8%)	38 (84.4%)	
Laparoscopy	22 (23.7%)	15 (31.2%)	7 (15.6%)	
Lymph node region				0.001
PLND-only	21 (22.6%)	4 (8.3%)	17 (37.8%)	
PLND+PAND	72 (77.4%)	44 (91.7%)	28 (62.3%)	
Lymphocyst				0.904
Yes	44 (47.3%)	23 (47.9%)	21 (46.7%)	
No	49 (52.7%)	25 (52.1%)	24 (53.3%)	
Number of surgeries				0.627
One	66 (71.0%)	33 (68.8%)	33 (73.3%)	
Two (restaging)	27 (29.0%)	15 (31.2%)	12 (26.7%)	
Chemotherapy				0.009
Yes	87 (93.5%)	48 (100.0%)	39 (86.7%)	
No	6 (6.5%)	0 (00.0%)	6 (13.3%)	

LNs, lymph nodes; FIGO, International Federation of Gynecology and Obstetrics; PLND, pelvic lymph node dissection; PLND+PAND, pelvic and para-aortic regions.

Based on the number of removed LNs, with the median number of nodes resected (24) serving as the cutoff, the two groups were defined as <24 and ≥24 LNs removed. No significant differences in age, FIGO stage, diameter of the tumor, presence of coexisting endometriosis, presence of ascites, peritoneal cytology, rupture of the capsule, surgical approach or the number of surgeries, and lymphocysts were observed between the two groups. However, for regions of LNs removed via the PLND with or without PAND, it was found that the proportion of patients who had ≥24 LNs removed through PLND+PAND was greater than that of those who had <24 LNs removed (*p* = 0.001). Moreover, the proportion of patients who received chemotherapy and had ≥24 LNs resected was significantly greater than that of those who received chemotherapy and had <24 LNs removed (*p* = 0.009). Only two patients received bevacizumab as part of their initial postoperative chemotherapy regimen among those who underwent surgery. Recurrent cases were observed in 15 patients, of whom six received treatment with bevacizumab.

**Table 2** presents the comparability of clinicopathological characteristics between patients with or without PAND. The two groups were not significantly different in terms of age, FIGO stage, diameter of the tumor, coexisting endometriosis, presence of ascites, peritoneal cytology, rupture of the capsule, surgical approach, number of surgeries, number of LNs dissected, or chemotherapy, and lymphocysts.

**Table 2:** Patient characteristics according to the region of removed lymph nodes.

Characteristics	Total N = 93	PLND-only N = 21	PLND+PAND N = 72	*p*-Value
Age (years)				0.586
Median (range)	49 (26–67)	48 (26–67)	49 (29–66)	
FIGO stage				0.809
IA	42 (45.2%)	9 (42.9%)	33 (45.8%)	
IC	51 (54.8%)	12 (57.1%)	39 (54.2%)	
Tumor diameter				0.943
≤15 cm	67 (72.0%)	15 (71.4%)	52 (72.2%)	
>15 cm	26 (28.0%)	6 (28.6%)	20 (27.8%)	
Coexisting endometriosis				0.232
Yes	51 (54.8%)	14 (66.7%)	37 (51.4%)	
No	42 (45.2%)	7 (33.3%)	35 (48.6%)	
Accompanied by ascites				0.815
Yes	33 (35.5%)	7 (22.6%)	26 (36.1%)	
No	60 (64.5%)	14 (66.7%)	46 (63.9%)	
Peritoneal cytology				0.585
Negative	86 (92.5%)	20 (95.2%)	66 (91.7%)	
Positive	7 (7.5%)	1 (4.8%)	6 (8.3%)	
Capsule rupture				0.564
Yes	48 (51.6%)	12 (57.1%)	36 (50.0%)	
No	45 (48.4%)	9 (42.9%)	36 (50.0%)	
Surgical approach				0.985
Laparotomy	71 (76.3%)	16 (76.2%)	55 (76.4%)	
Laparoscopy	22 (23.7%)	5 (23.8%)	17 (23.6%)	
The number of lymph nodes				0.092
≥24 LNs removed	48 (51.6%)	4 (19.0%)	28 (38.9%)	
<24 LNs removed	45 (48.4%)	17 (81.0%)	44 (61.1%)	
Lymphocyst				0.336
Yes	44 (47.3%)	8 (38.1%)	36 (50.0%)	
No	49 (52.7%)	13 (61.9%)	36 (50.0%)	
Number of surgeries				0.298
One	66 (71.0%)	13 (61.9%)	53 (73.6%)	
Two (restaging)	27 (29.0%)	8 (38.1%)	19 (26.4%)	
Chemotherapy				0.720
Yes	87 (93.5%)	20 (95.2%)	67 (93.1%)	
No	6 (6.5%)	1 (4.8%)	5 (6.9%)	

PLND, pelvic lymph node dissection; PLND+PAND, pelvic and para-aortic regions; FIGO, International Federation of Gynecology and Obstetrics; LNs, lymph nodes.

In general, a higher number of LNs removed is associated with increased complications. Based on the surgical approach, patients were categorized into the following two groups: laparotomy and laparoscopy (**Table 3**). In the laparotomy and laparoscopy groups, the median intraoperative blood loss was 400 mL and 200 mL, respectively. The mean operative time for the two groups was recorded as 262.99 and 334.00 min. Additionally, the mean hospitalization day for these groups was found to be 8.89 days and 7.71 days, respectively. In the laparotomy group, 13 (18.3%) patients received a blood transfusion, while in the laparoscopy group, only one (4.5%) patient received a blood transfusion. Regarding intensive care unit (ICU) admission, 17 (23.9%) patients in the laparotomy group and four (18.2%) in the laparoscopy group required postoperative ICU admission. Lymphocyst formation emerged as a common complication observed among our study participants following lymphadenectomy. In this study, a total of 44 (47.3%) patients developed lymphocysts; there were 36 (50.7%) patients diagnosed with lymphocysts in the laparotomy group, whereas the number of patients with lymphocysts in the laparoscopy group was eight (33.4%). Although most of these patients remained asymptomatic, two patients developed infected lymphocysts requiring incision and drainage. In general, autonomous reabsorption of lymphocysts can occur without intervention. Wound fat liquefaction was observed in only two (2.8%) patients within the laparotomy group in terms of postoperative healing of incisions. Only one patient developed a postoperative pulmonary infection in the laparoscopy group. Significantly higher levels of intraoperative blood loss (*p* = 0.003) and operation time (*p* = 0.027) were observed in the laparotomy group compared to the laparoscopy group. There were no statistically significant differences in the length of hospitalization day, blood transfusion, ICU admission, lymphocyst, wound fat liquefaction, and pulmonary infection between the two groups.

**Table 3:** Complications of patients with lymphadenectomy.

Complications	Total N = 93	Laparotomy N = 71	Laparoscopy N = 22	*p*-Value
Intraoperative blood loss (mL)				0.003
Median	300	400	200	
Operative time (min)				0.027
Mean	279.79	262.99	334.00	
Hospitalization time (day)				0.095
Mean	8.66	8.89	7.91	
Blood transfusion				0.175
Yes	14 (15.1%)	13 (18.3%)	1 (4.5%)	
No	79 (84.9%)	58 (81.7%)	21 (95.5%)	
ICU admission				0.772
Yes	21 (22.6%)	17 (23.9%)	4 (18.2%)	
No	72 (77.4%)	54 (76.1%)	18 (81.8%)	
Lymphocyst				0.329
Yes	44 (47.3%)	36 (50.7%)	8 (33.4%)	
No	49 (52.7%)	35 (49.3%)	14 (63.6%)	
Wound fat liquefaction				1.000
Yes	2 (2.2%)	2 (2.8%)	0 (0.0%)	
No	91 (97.8%)	69 (97.2%)	22 (100.0%)	
Pulmonary infection				0.237
Yes	1 (1.1%)	0 (0.0%)	1 (4.5%)	
No	92 (98.9%)	71 (100.0%)	21 (95.5%)	

ICU, intensive care unit.

### Survival analysis

With a median follow-up of 78 (range, 11–142) months, 15 (16.1%) patients developed tumor recurrence—five underwent resection of ≥24 LNs and 10 with <24 LNs removed. The pelvic and abdominal cavities were the most common sites of disease recurrence.

**Figure 1:**
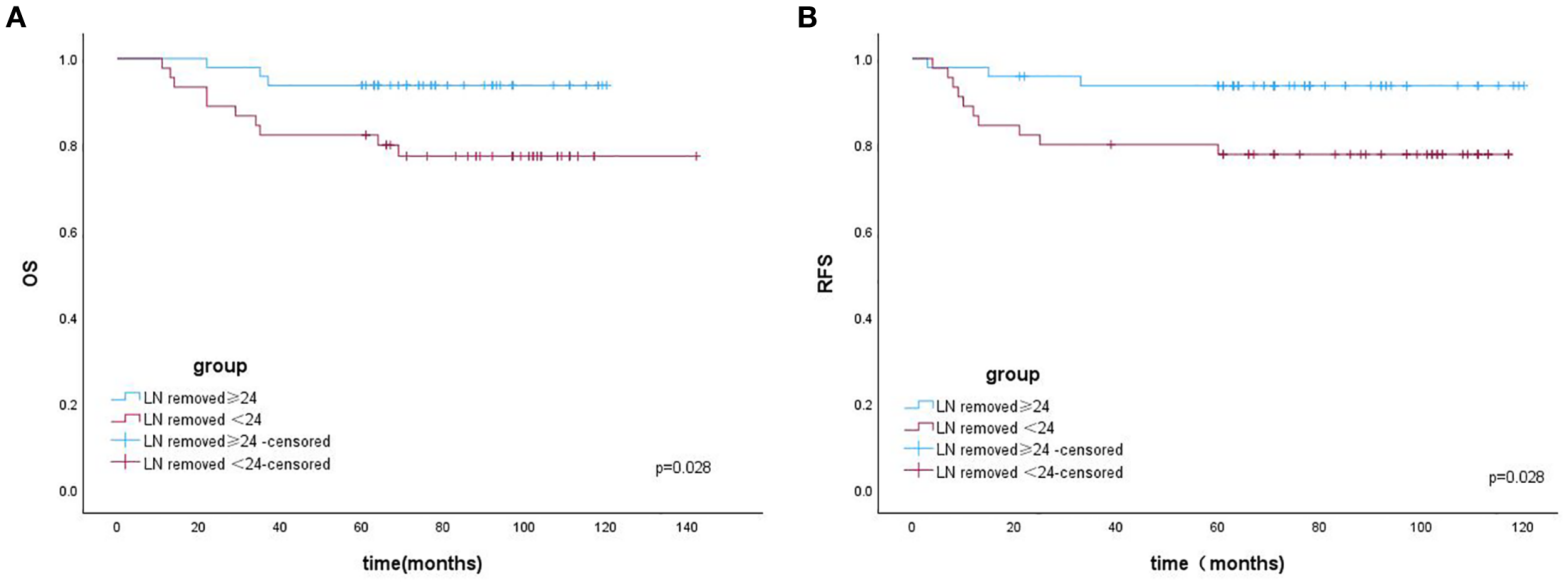
**(A)** The overall survival of patients with OCCC according to the number of removed LNs. **(B)** The recurrence-free survival of patients with OCCC according to the number of removed LNs.

**Figure 2:**
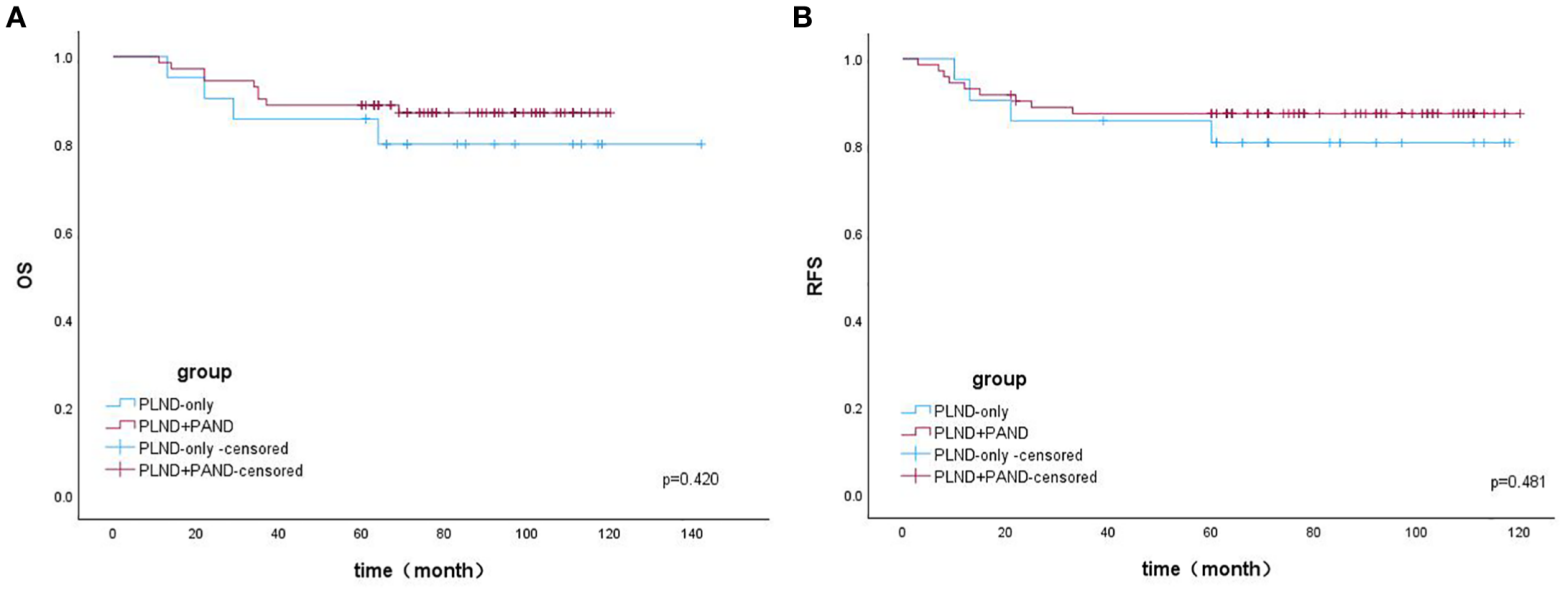
**(A)** The overall survival of patients with OCCC according to the region of removed LNs. **(B)** The overall survival of patients with OCCC according to the number of removed LNs.

The Kaplan–Meier (K–M) curves according to the number of removed LNs are illustrated in **Figures 1A, B**. Patients who underwent resection of ≥24 LNs had significantly higher OS (log-rank test, *p* = 0.028) and RFS (log-rank test, *p* = 0.028) than those who underwent resection of <24 LNs. The K–M curves for the regions of removed LNs are shown in **Figures 2A, B**. No difference was noted in OS (log-rank test, *p* = 0.420) or RFS (log-rank test, *p* = 0.481) between the PLND-only and PLND+PAND groups.

**Table 4** presents the results of the Cox regression analysis for the prognostic factors for RFS. Univariate analysis demonstrated that younger age [hazard ratio (HR) = 1.07; 95% confidence interval (CI) = 1.0–1.14; *p* = 0.031] and the higher number of removed LNs (HR = 3.85; 95% CI = 1.06–13.98; *p* = 0.041) were significantly and independently related to improved outcomes; however, positive peritoneal cytology (HR = 5.11; 95% CI = 1.40–18.63; *p* = 0.013) was significantly and independently related to poor outcomes.

**Table 4:** Univariate and multivariate analyses for factors associated with RFS.

Characteristics	Total No. (%)	Univariate			Multivariate		
		HR	95% CI	*p*-Value	HR	95% CI	*p*-Value
Age (years)							
≤Median	49 (52.7%)	1.00	-	-			
>Median	44 (47.3%)	1.07	1.0–1.14	0.031			
FIGO stage							
IA	42 (45.2%)	1.00	-	-			
IC	51 (54.8%)	1.93	0.59–6.26	0.275			
Tumor diameter							
≤15 cm	67 (72.0%)	1.00	-	-			
>15 cm	26 (28.0%)	2.38	0.80–7.07	0.120			
Peritoneal cytology							
Negative	86 (92.5%)	1.00	-	-			
Positive	7 (7.5%)	5.11	1.40–18.63	0.013			
Capsule rupture							
Yes	48 (51.6%)	1.00	-	-			
No	45 (48.4%)	1.31	0.44–3.89	0.630			
Coexisting endometriosis							
Yes	51 (54.8%)	1.00	-	-			
No	42 (45.2%)	0.72	0.24–2.20	0.562			
Surgical approach							
Laparotomy	71 (76.3%)	1.00	-	-			
Laparoscopy	22 (23.7%)	0.26	0.03–1.99	0.195			
Number of surgeries							
One	66 (71.0%)	1.00	-	-	1.00	-	-
Two (restaging)	27 (29.0%)	1.58	0.52–4.83	0.424	8.35	1.69–41.24	0.009
Number of nodes resected							
≥24	48 (51.6%)	1.00	-	-	1.00	-	-
<24	45 (48.4%)	3.85	1.06–13.98	0.041	9.49	1.69–53.43	0.011
Region of lymphadenectomy							
PLND-only	21 (22.6%)	1.00	-	-			
PLND+PAND	72 (77.4%)	0.67	0.21–2.16	0.499			
Chemotherapy							
Yes	87 (93.5%)	1.00	-	-			
No	6 (6.5%)	0.05	0–561.96	0.519			

RFS, recurrence-free survival; HR, hazard ratio; FIGO, International Federation of Gynecology and Obstetrics; PLND, pelvic lymph node dissection; PLND+PAND, pelvic and para-aortic regions.

Restage surgery (HR = 8.35; 95% CI = 1.69–41.24; *p* = 0.009) and a lower number of removed LNs (HR = 9.49; 95% CI = 1.69–53.43; *p* = 0.011) were significantly related to poor outcomes as revealed through multivariate analysis.

**Table 5** displays the results of the Cox regression analysis for the prognostic factors for OS. Younger age (HR = 1.07; 95% CI = 1.0–1.14; *p* = 0.028) and a higher number of removed LNs (HR = 3.81; 95% CI = 1.05–13.87; *p* = 0.042) were significantly and independently related to improved outcomes as revealed through univariate analysis; however, positive peritoneal cytology (HR = 4.42; 95% CI = 1.21–16.15; *p* = 0.025) was significantly and independently related to poor outcomes.

**Table 5:** Univariate and multivariate analyses for factors associated with OS.

Characteristics	Total No. (%)	Univariate			Multivariate		
		HR	95% CI	*p*-Value	HR	95% CI	*p*-Value
Age (years)							
≤Median	49 (52.7%)	1.00	-	-			
>Median	44 (47.3%)	1.07	1.0–1.14	0.028			
FIGO stage							
IA	42 (45.2%)	1.00	-	-			
IC	51 (54.8%)	1.90	0.58–6.16	0.287			
Tumor diameter							
≤15 cm	67 (72.0%)	1.00	-	-			
>15 cm	26 (28.0%)	2.23	0.75–6.65	0.149			
Peritoneal cytology							
Negative	86 (92.5%)	1.00	-	-			
Positive	7 (7.5%)	4.42	1.21–16.15	0.025			
Capsule rupture							
Yes	48 (51.6%)	1.00					
No	45 (48.4%)	1.29	0.43–3.82	0.653			
Coexisting endometriosis							
Yes	51 (54.8%)	1.00	-	-			
No	42 (45.2%)	0.72	0.24–2.20	0.560			
Surgical approach							
Laparotomy	71 (76.3%)	1.00	-	-			
Laparoscopy	22 (23.7%)	0.27	0.04–2.09	0.211			
Number of surgeries							
One	66 (71.0%)	1.00	-	-	1.00	-	-
Two (restaging)	27 (29.0%)	1.60	0.52–4.90	0.409	6.21	1.45–26.62	0.014
Number of nodes resected							
≥24	48 (51.6%)	1.00	-	-	1.00	-	-
<24	45 (48.4%)	3.81	1.05–13.87	0.042	5.85	1.30–26.27	0.021
Region of lymphadenectomy							
PLND-only	21 (22.6%)	1.00	-	-			
PLND+PAND	72 (77.4%)	0.62	0.19–2.01	0.426			
Chemotherapy							
Yes	87 (93.5%)	1.00	-	-			
No	6 (6.5%)	0.05	0–545.56	0.517			

OS, overall survival; HR, hazard ratio; FIGO, International Federation of Gynecology and Obstetrics; PLND, pelvic lymph node dissection; PLND+PAND, pelvic and para-aortic regions.

Multivariate analysis demonstrated that restage surgery (HR = 6.21; 95% CI = 1.45–26.62; *p* = 0.014) and a lower number of removed LNs (HR = 5.85; 95% CI = 1.30–26.27; *p* = 0.021) were significantly related to poor outcomes.

## Discussion

EOC is a malignancy occurring in women that is often associated with high tumor load. Improved survival rates have been shown in patients with EOC, where complete resection is defined as no visible residual disease (11, 12). An important prognostic factor impacting the OS for EOC is the removal of all visible tumors (13). Even in advanced-stage EOC, the challenge of the decision-making process for patients with high risk for surgery with curative intent versus palliative treatment is owing to the balance between an expected improved survival, if complete debulking is achieved, and the expected surgical morbidity and mortality (13). For early-stage EOC, complete surgical staging is particularly significant; lymphadenectomy is an important measure of complete surgical staging. The therapeutic significance of lymphadenectomy for treating patients with early-stage EOC has been debatable for two decades. Several conflicting opinions exist regarding the impact of lymphadenectomy on early-stage EOC (9, 14–19).

A randomized study conducted by Maggioni et al. (14) among patients with early-stage EOC revealed a non-statistically significant effect of systematic lymphadenectomy on RFS or OS; however, it can correctly estimate progression or death from any cause-favored lymphadenectomy. Systematic lymphadenectomy seems to be a relatively safe and acceptable surgical procedure when performed in selected gynecologic oncology institutions and can guarantee the optimal accuracy of staging, which allows for tailoring postoperative treatments. Deng et al. (15) suggested that LN dissection was associated with an increased incidence of perioperative adverse events; however, it was not associated with a gain in OS or RFS. Suzuki et al. (16) analyzed the role of lymphadenectomy in more than 200 patients with pTI-IIB (FIGO stage IA–IIB) OCCC. Their study data suggested no significant difference in the OS and RFS in FIGO stage IA–IIB OCCC regardless of the completion of surgical staging lymphadenectomy. A multicentric randomized trial assessing the efficacy of systematic lymphadenectomy in early-stage EOC demonstrated no statistically significant difference in a 5-year OS (84.0% vs. 81.6%) between the lymphadenectomy and control groups. However, a reduced risk of recurrence was observed in patients undergoing systematic nodal dissection from 30% to 22% (14). Although no first-level evidence exists suggesting survival benefits of lymphadenectomy in early-stage EOC, clinicians can detect macroscopic nodal disease through systematic lymphadenectomy and can identify patients who will benefit from adjuvant treatments. Unnecessary postoperative chemotherapy should be avoided in low-risk confined diseases. A thorough evaluation of the pros and cons of systematic lymphadenectomy is critical, considering surgery-related morbidity, relative costs, and the absence of overall survival benefits. Therefore, before deciding whether to perform this procedure, it is essential to analyze and discuss with each patient with early EOC (20). Chen et al. (17) demonstrated that systematic LN dissection may be discarded in patients with apparent early-stage low-grade mucinous and endometrioid EOC; however, it may be considered for patients with apparent early-stage low-grade serous OCCC. According to the histological subtype, the highest incidence of LN metastasis has been found in the serous subtype (23.3%), followed by clear cells (14.4%) and endometrioid (6.5%), and the lowest in the mucinous subtype (2.6%) (21). OCCC exhibits a histological subtype characterized by a relatively elevated propensity for LN metastasis. Yamazaki et al. (9) suggested that full lymphadenectomy should be regularly investigated in patients with OCCC at potential risk of LN metastasis. The nodal spreading pattern in EOC may differ across histological types. Therefore, we determined the effect of the number and region of lymphadenectomy on the survival of patients with stage I OCCC.

A poor response to conventional chemotherapy is observed in patients with OCCC, thus increasing the importance of surgical staging, including retroperitoneal lymphadenectomy. Moreover, the benefit of adequate lymphadenectomy is that it reduces the chance of diagnosing an advanced-stage disease when a greater number of LNs are removed by identifying occult metastasis (stage shifting). For stage I OCCC, adequate lymphadenectomy is critical. However, how can the “adequate” lymphadenectomy be defined? The standard lymphadenectomy included systematic pelvic and para-aortic lymphadenectomy up to the level of the renal vessel or inferior mesenteric artery; this was the only procedure that covered all regional LNs. However, the number of removed LNs was not specified. Although the quality and extent of systematic lymphadenectomy may vary depending on the gynecologist who performs the surgery and the anatomical variants among patients, the number of removed LNs was relatively objective based on the pathology results. In this study, when ≥24 LNs were removed, patients underwent PLND+PAND. Therefore, adequate lymphadenectomy should also include the number of LNs dissected.

In the study by Maggioni et al. (8), bilateral PLND and aortic LN dissection were regarded as satisfactory when at least 20 and 15 nodes were removed, respectively. In a nationwide cohort study, Kleppe et al. (18) suggested that adequate lymphadenectomy should be performed when at least 10 LNs have been removed; however, it is preferable when >20 LNs have been removed for those with early-stage ovarian carcinoma. Matsuo et al. (19) reported that resection of at least 8–12 LNs is sufficient to identify one nodal metastasis in early-stage ovarian carcinoma. Moreover, lymphadenectomy for different histological subtypes was examined, and adequate lymphadenectomy was reported to be associated with survival in patients with ovarian clear cell tumors. Our study demonstrated that the cutoff value for the number of removed LNs was 24, and patients with ≥24 removed LNs had better RFS and OS than those with <24 removed LNs. These results suggested that removing more LNs leads to accurate staging and improves the prognosis for patients with early-stage OCCC.

The significance of adequate lymphadenectomy for early-stage OCCC has been widely reported (7, 9, 10, 22, 23). Two of these studies focused on the number of LNs involved in early-stage OCCC (7, 10). Mahdi et al. (7) concluded that among clinical early-stage OCCC patients without LN metastasis, those with 11 or more LNs removed tended to have better RFS and OS than those with 1–10 LNs removed. Takei et al. (10) demonstrated that having ≥35 LNs removed was an independent predictor of improved RFS in patients with stage I OCCC, and sufficient lymphadenectomy may improve the prognosis for such patients. Although the number of removed LNs differed, the number of lymphadenectomies was an independent prognostic factor.

Our study has several limitations. First, this study inevitably had selection bias owing to its retrospective nature and being conducted at a single institution. Our study primarily analyzed patients in stage I diagnosed by postoperative pathology, thereby excluding those with upstage or LN metastasis. Second, in comparison to multicenter studies, in which the number of patients was too small to reach conclusive results, our findings should be verified in a larger study. Efforts should be made to conduct a multicenter study in the future. Although the difference in the number of resected LNs may be because of the degree of intraabdominal adhesion and distribution and tortuosity of the blood vessels, these factors could not be assessed in this study because they were not included in the medical records. Additionally, the study’s database did not contain much information regarding the sequelae of lymphadenectomy, such as lymphedema or surgical complications related to lymphadenectomy.

## Conclusions

In conclusion, this study demonstrated that the number of removed LNs, as an important part of adequate lymphadenectomy for stage I OCCC, contributed to better RFS and OS. The number of dissected LNs was an independent prognostic factor for stage I OCCC. Sufficient lymphadenectomy may improve the survival of patients with stage I OCCC.

## Data Availability

The original contributions presented in the study are included in the article/Supplementary Material. Further inquiries can be directed to the corresponding author.
